# Design and Development of Neomycin Sulfate Gel Loaded with Solid Lipid Nanoparticles for Buccal Mucosal Wound Healing

**DOI:** 10.3390/gels8060385

**Published:** 2022-06-16

**Authors:** Khaled M. Hosny, N. Raghavendra Naveen, Mallesh Kurakula, Amal M. Sindi, Fahad Y. Sabei, Adel Al Fatease, Abdulmajeed M. Jali, Waleed S. Alharbi, Rayan Y. Mushtaq, Majed Felemban, Hossam H. Tayeb, Eman Alfayez, Waleed Y. Rizg

**Affiliations:** 1 Department of Pharmaceutics, Faculty of Pharmacy, King Abdulaziz University, Jeddah 21589, Saudi Arabia; kmhomar@kau.edu.sa (K.M.H.); wsmalharbi@kau.edu.sa (W.S.A.); wrizq@kau.edu.sa (W.Y.R.); 2 Center of Excellence for Drug Research and Pharmaceutical Industries, King Abdulaziz University, Jeddah 21589, Saudi Arabia; 3 Department of Pharmaceutics, Sri Adichunchanagiri College of Pharmacy, Adichunchanagiri University, B.G. Nagar 571448, Karnataka, India; raghavendra.naveen@gmail.com; 4 Product Development Department, CURE Pharmaceutical, Oxnard, CA 93033, USA; 5 Department of Oral Diagnostic Science, Faculty of Dentistry, King Abdulaziz University, Jeddah 21589, Saudi Arabia; amsindi@kau.edu.sa; 6 Department of Pharmaceutics, College of Pharmacy, Jazan University, Jazan 45142, Saudi Arabia; fsabei@jazanu.edu.sa; 7 Department of Pharmaceutics, College of Pharmacy, King Khalid University, Abha 62529, Saudi Arabia; afatease@kku.edu.sa; 8 Department of Pharmacology and Toxicology, College of Pharmacy, Jazan University, Jazan 82511, Saudi Arabia; amjali@jazanu.edu.sa; 9 Department of Pharmaceutics, College of Clinical Pharmacy, Imam Abdulrahman Bin Faisal University, Dammam 31441, Saudi Arabia; rymushtaq@iau.edu.sa; 10 Department of Medical Laboratory Sciences, Faculty of Applied Medical Sciences, King Abdulaziz University, Jeddah 21589, Saudi Arabia; maafelemban1@kau.edu.sa; 11 Centre for Artificial Intelligence in Precision Medicines, King Abdulaziz University, Jeddah 21589, Saudi Arabia; 12 Nanomedicine Unit, Center of Innovation in Personalised Medicine, King Abdulaziz University, Jeddah 21589, Saudi Arabia; hhtayeb@kau.edu.sa; 13 Department of Oral Biology, Faculty of Dentistry, King Abdulaziz University, Jeddah 21589, Saudi Arabia; ealfayez@kau.edu.sa

**Keywords:** health care, neomycin sulfate, solid lipid nanoparticles, optimization, gels, sustainability of natural resources, wound healing

## Abstract

Drug administration to the wound site is a potential method for wound healing. The drug retention duration should be extended, and drug permeability through the buccal mucosal layer should be regulated. Oral wounds can be caused by inflammation, ulcers, trauma, or pathological lesions; if these wounds are not treated properly, they can lead to pain, infection, and subsequent undesirable scarring. This study aimed to develop Kolliphor-407 P-based gel containing neomycin sulfate (NES) loaded in solid lipid nanoparticles (SLNs) and enhance the antimicrobial activity. By considering lipid concentrations and achieving the lowest particle size (Y1) and maximum entrapment (EE-Y2) effectiveness, the formulation of NES-SLN was optimized using the Box–Behnken design. For the selected responses, 17 runs were formulated (as anticipated by the Design-Expert software) and evaluated accordingly. The optimized formulation could achieve a particle size of 196.25 and EE of 89.27% and was further utilized to prepare the gel formulation. The NES-SLN-G formula was discovered to have a smooth, homogeneous structure and good mechanical and rheological properties. After 24 h of treatment, NES-SLN-G showed a regulated in vitro drug release pattern, excellent ex vivo permeability, and increased in vitro antibacterial activity. These findings indicate the potential application of NES-SLN-loaded gels as a promising formulation for buccal mucosal wound healing.

## 1. Introduction

Wound healing is a dynamic and complex process involving the replacement of devitalized cellular structures and tissue layers. A successful wound dressing should keep the site moist, allow for gas exchange, act as a microbial barrier, and eliminate excess exudates [1]. Oral wounds can be caused by trauma, recurrent ulcers, inflammation, irradiation, and surgery for the extirpation of congenital or pathological lesions. If not properly treated, intraoral wounds can lead to pain, infection, and subsequent undesirable scarring and adhesion, resulting in functional deficits, such as dysphagia, dysarthria, and a poor quality of life. Advanced dressings, such as hydrocolloid foam and hydrogel, provide a moist environment for wounds today [2].

Hydrogel is a three-dimensional (3D) network made up of hydrophilic polymer chains that can hold abundant water in their structures, allowing it to be used as a drug carrier system [3] and wound dressing [4,5]. Various wound dressings have recently been explored, although certain defects have been discovered, such as poor mechanical qualities and low fluid absorption capability [6,7]. Furthermore, because antibiotics might speed up wound healing [8,9], neomycin sulfate (NES) was included as a medication in this hydrogel dressing. The aminoglycoside antibiotic NES has a broad range and has been used topically on the skin and mucous membranes [10,11].

Solid lipid nanoparticles (SLNs) are colloidal lipid nanocarriers with a solid core. They are one of the ideal medication delivery devices since they are made of biocompatible materials [12]. SLNs can improve the loading of both water- and fat-soluble medicines. SLNs have several advantages over other drug delivery systems, including being biocompatible and biodegradable with low toxicity, enabling a regulated or sustained drug release profile, and having high protective and loading capacities with minimum adverse effects [13,14]. SLNs have previously been shown to improve valsartan transdermal penetration for systemic antihypertensive action [15].

Furthermore, SLNs are an effective dermal drug delivery method, with improved dermal penetration, occlusive properties, and a longer residence duration inside the skin layers [16]. The integration of SLNs in gels has previously been shown to result in higher drug permeability than standard gel formulations, which has been attributed to the close interaction of SLNs with the stratum corneum, resulting in a longer residence time in skin layers [17]. As a result, there has been a lot of focus in recent years on developing SLN-based formulations for topical medication administration.

Pharmaceutical development is a lengthy and complex process that begins with the formulation and ends with the finished product. In any case, the pharmaceutical product should provide the patient with a certain level of quality. Despite advances in pharmaceutical technology and medicine manufacturing, the pharmaceutical industry still relies heavily on traditional process development and drug manufacturing methods. The classic formulation development technique employs raw material controls, product manufacturing controls, in-process controls, and final product controls to ensure product quality.

As a result, detecting the link between product features and product quality using an empirical technique is challenging, and quality is not always guaranteed [18]. International initiatives established the idea of Quality by Design (QbD), and regulatory organizations such as the FDA and EMA have forcefully encouraged the pharmaceutical industry to utilize it. The International Conference on Harmonization (ICH) published significant recommendations in Q8 Pharmaceutical Development, Q9 Quality Risk Management, and Q10 Pharmaceutical Quality System, which defined the concepts and use of the QbD approach. In summary, QbD is a systematic, scientific, risk-based method for formulating and refining manufacturing processes to achieve a set quality for the final product. The idea’s primary motivation, according to QbD, is that “quality cannot be tested into items; it should be built-in or by design” [19,20]. This study’s primary goal was to combine cutting-edge technologies for formulation development, such as nanoparticle technology and QbD. The study was also performed using a modified solvent injection approach to manufacture lipid nanoparticles, followed by the construction of a suitable lipid nanoparticle-containing gel formulation.

## 2. Results and Discussion

### 2.1. Compatibility Studies

**Figure 1:**
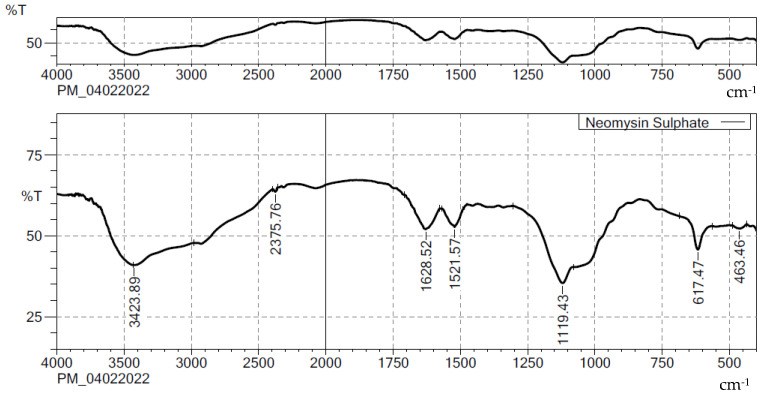
FTIR spectra of NES.

**Figure 2:**
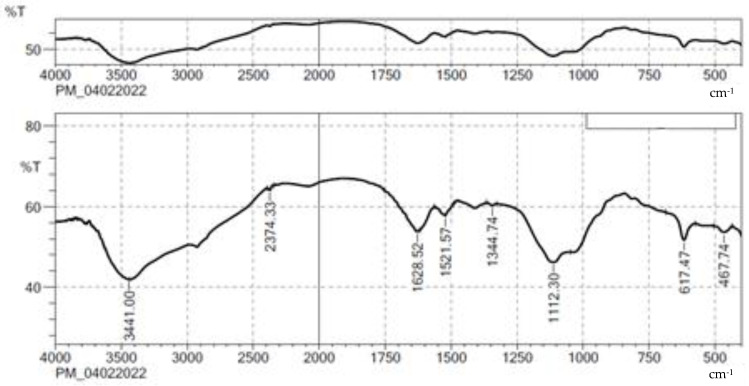
FTIR spectra of physical mixture of NES + all ingredients.

The characteristic band of NES and the physical mixture of optimized formulation ingredients are shown in Figure 1 and Figure 2, respectively. The bands at 3423.89, 3782.24, and 2348.74 cm^−1^ were due to NH stretching, OH stretching, and C-H stretching (aromatic), respectively. C=C stretching and C-H stretching were observed at 1521.57 and 3898.75 cm^−1^, and 1628.52 and 1119.43 cm^−1^ correspond to C-O and C=O stretching. In the end, these bands were identified in the formula. The spectrum revealed that pure NES and selected ingredients were mutually compatible. 

### 2.2. Optimization of Preparation of NES-SLN

The optimal degree of the parameters chosen and their interconnection in achieving desirable EE and mucoadhesion qualities was evaluated using the Box–Behnken design of response surface methodology (RSM). Table 1 summarizes the results of 17 different experimental runs. EE was between 72 and 89 percent for all trial preparations, with particle sizes ranging from 201 to 398. Individual responses were examined, and statistical modeling, such as ANOVA and fx, was used to determine the effects of parameters.

The quadratic model was chosen for all responses based on the sum of squares (Type I) and fit summary (adjusted and projected R2) (Table 2). A quadratic model (high-order polynomial) was chosen since the auxiliary terms are visible and the model does not alias. The predicted R2 values of 0.9130 and 0.9628 agree with the adjusted R2 of 0.9730 and 0.9639, respectively, because the difference is smaller than 0.2. Adequate precision measures the S/N ratio. A fraction greater than four is usually preferred. The particle size ratio to EE was 27.5567 to 26.0962, showing a relevant signal. As a result, the model’s efficiency in operating the design space is confirmed. The model F-values of both responses were 65.10 and 48.51, respectively, indicating that the model is valid. Only a 0.01 percent possibility exists that the high F-value was caused by noise.

The model’s repeatability is indicated by the coefficient of variation (CV) value. The current model’s repeatability was determined to be CV 10%. The investigation found relatively low CV values, indicating that the model is accurate and reliable. Insufficient fit can result in a model that fails to depict all of the data (Table 3). As a result, determining that the model-generated equations predict the results rationally requires a lack of fit. The chosen model was appropriate for the investigation because the *p*-values for both responses were insignificant [21].

The inference of the quantitative impacts of the factor components was tested using ANOVA. Multiple regressions were used to extract polynomial equations from the data. [22]. The ANOVA results outperformed the quadratic equation’s statistical significance; the *p*-value was 0.0500, indicating the importance of model terms. The test design stipulated that particle size was primarily influenced by (a) antagonistic effects of A, AB, AC, and BC and the polynomial term of A, with *p*-values of 0.0031, 0.0001, 0.0441, and 0.0175; (b) synergistic effects of B and C and polynomial terms of B and C, with *p*-values of 0.0169, 0.0017, 0.0086, and 0.0001, respectively, with (i) an antagonist effect of polynomial terms of B and C with *p*-values of 0.0001 and 0.0003; (ii) a synergistic effect of B, C, AB, and AC and the polynomial term of A, with *p*-values of 0.0072, 0.0003, 0.0001, 0.0001, and 0.0018, respectively, and the term AC affected EE with a high magnitude among the essential variables. Table 4 shows the ANOVA coefficients and their *p*-values for both solutions. The following are the equations obtained from the responses for the best possible model:

Particle size = +264.20 − 14.37 A + 10.13 B + 16.00 C − 54.50 AB − 69.75 AC − 11.25 BC − 13.85 A^2^ + 16.15 B^2^ + 51.40 C (1)

EE = +83.20 + 0.3750 A + 1.13 B + 2.00 C + 4.25 AB + 4.50 AC − 1.0000 BC +2.02 A^2^ − 3.98 B^2^ − 2.72 C^2^ (2)

**Figure 3:**
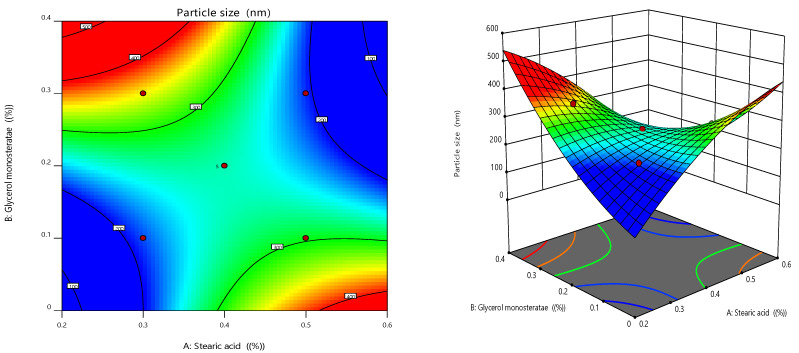

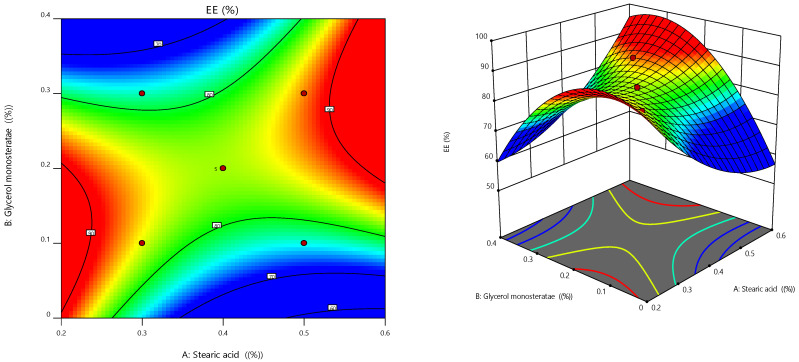
Response surface graphs for EE and in vitro mucoadhesion (3-dimensional and contour).

**Figure 4:**
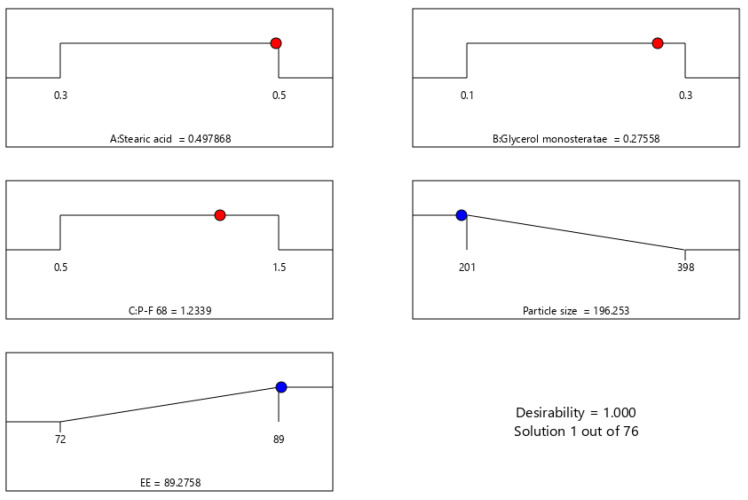
Desirability bar graph for the optimized result.

RSM also looked into and presented the effect of individual modifiers on responses [23]. Figure 3 depicts the link between the response and the variables and a contour plot depicting the variable influences. RSM was used to determine and explain the effect of non-dependent variables on the resulting individual responses. Three-dimensional response surface graphs are required to demonstrate the interaction and primary effect. The acquired solutions are displayed using contour plots [24]. The global desirability (D) function optimized the model order. Every response was limited to the maximum to obtain an inlay graph to augment the non-dependent variables. All three possible independent variables were encompassed in the design for optimization [25]. The independent variables (ideal level) in the desirability function plot in Figure 4 reflected a maximum desirability value of 1.000 for both responses. As a consequence, the particle size is 196.253 nm, and the EE is 89.275 when utilizing this setting. These altered concentrations were used to construct the O-NES-SLN formulation and subsequently used to make gels. The optimization result was confirmed by creating a formulation based on the optimization results and comparing the experimental results. The relative error was less than 2%, indicating that the experiment was successful [26].

### 2.3. Formulation and Characterization of NES-SLN-G

In support of the optimization findings, a gel formulation prepared using NES-SLN revealed a particle size of 196.5 ± 1.5 nm. Because of the decreased interfacial tension, particle size decreases with increasing surfactant content, facilitating miscibility between the layers of SLN dispersions. Due to the production of micelles, any subsequent rise in concentration results in a rising trend in particle size. The surface becomes fully loaded with surfactant molecules at the critical micelle concentration. Thus, the interfacial tension change is virtually minimal beyond the critical micelle concentration, resulting in larger particle sizes. The type of surfactants and the lipid components of SLNs are the two key components that can affect drug encapsulation efficiency. With increasing surfactant concentrations at constant lipid concentrations, drug content and encapsulation efficiency improved [27]. The partition phenomenon, characterized by increased drug partitioning from the inner to outer phase due to the presence of a high surfactant concentration in the exterior phase, which supports increased drug leakage from the internal to external phase, could also explain the decreased entrapment efficiency. The NES-SLN-G polydispersity index was found to be 0.15. The particle size of pure NES in a gel formulation was 542.5 ± 4.2 nm, with a polydispersity index (PDI) of 0.58 Table 5. In general, the numerical value of PDI ranges from 0.0 (for a perfectly uniform sample concerning particle size) to 1.0 (for a perfectly uniform sample with respect to particle size) (for a highly polydisperse sample with multiple particle size populations). In reality, polymer-based nanoparticle materials with values of 0.2 and below are usually deemed acceptable. The average zeta potential of NES-SLN-G was determined to be −32.5 mV, indicating that the formulation is stable. This negative potential has been attributed to glyceryl monostearate, a fatty acid ester.

**Figure 5:**
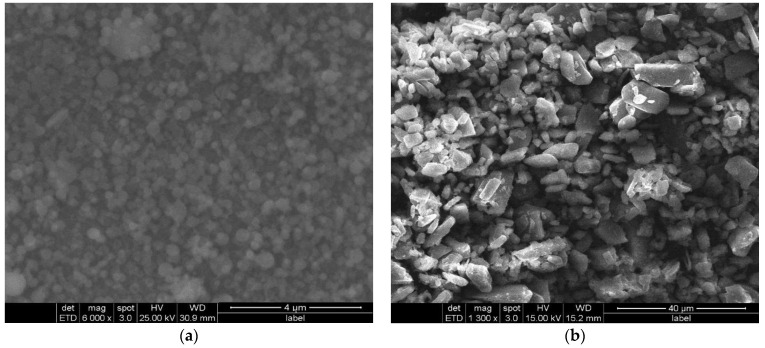
SEM of (**a**) NES-SLN-G and (**b**) NES-G formulations.

SEM is a typical technique for evaluating surface morphology and nanoformulation properties. A scanning electron microphotograph of both the gel formulations revealed that NES-SLN-G was uniformly formed and had a well-defined perimeter [28]. In addition, there was no visible agglomeration of the lipid nanoparticles in the SEM images, indicating consistent dispersion of the formulation (Figure 5). NES-G showed variable sizes and forms and nonuniformity in the size distribution, in contrast to the basic gel formulation. For all of the samples, the size of the lipid nanoparticles was found to be consistent with the dynamic light scattering data. Particle size and shape are critical in nanotechnology-based formulations because they affect physical features such as texture and flowability and medicinal efficacy.

The NES-SLN-based gel had a smooth, uniform composition with a pH of 5.85 ± 0.15, making it appropriate for topical use. The final gel formulation’s viscosity was determined to be 92,105 mPa∙s. Similarly, the value of spreadability was determined to be 5.92 g.cm/sec, indicating that the gel could be spread easily with minimal shear. Even with a blank gel, the same findings were obtained. Surface tension governs the interactions between two immiscible substances. With increasing surfactant concentration, the surface tension of the SLNs usually decreases. The higher surfactant content reduces the surface area of each particle in the formulation by reducing the superficial tension. The presence of optimized surfactant concentration with additional surfactant properties of glyceryl monostearate was found to have a minimum surface tension of 20.52 ± 1.52 dynes/cm^2^, which can be attributed to the additional surfactant properties of glyceryl monostearate, which likely increases the retention and permeation of the NES. The specific gravity [0.997 ± 0.04 and 0.995 ± 0.06] and density [0.996 ± 0.05 and 0.998 ± 0.03] of the NES-SLN-G and NES-G gel formulations were similar to those of deionized water.

### 2.4. Ex Vivo Drug Release Studies

**Figure 6:**
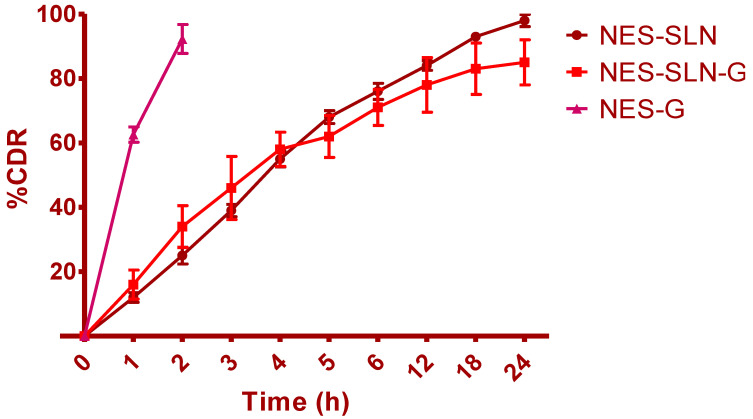
Ex vivo drug release of NES-G, NES-SLN-G, and NES-SLN (Avg ± S.D; *n* = 6) (NES concentration of 0.5%, pH 5.5 at 32 ± 2 °C).

NES release, a valuable indicator of in vivo drug performance, was examined using a modified Franz diffusion cell at a skin temperature of 32+− 2 °C. The Franz diffusion cell had a cellulose acetate membrane with a pore size of 0.45 m clamped between the donor and recipient compartments. The drug release from the SLNs was biphasic, with a burst of drug release followed by regulated drug release (Figure 6). The drug’s first abrupt release could be owing to the drug’s availability in the interphase and hydrophilic phases of SLNs. By the end of 24 h, almost 97.25 percent of NES had been released. The NES-SLN-G formulation demonstrated substantially faster NES release until the end of 4 h, followed by controlled release. Due to increased viscosity and density after forming into gels, medication release was significantly delayed after 4 h. Only 84.78 percent of the NES had been released by the end of the study. By the end of just 2 h, a blank gel formulation containing simple NES had shown very rapid and complete release.

### 2.5. In Vitro Antimicrobial Activity Assessment

**Figure 7:**
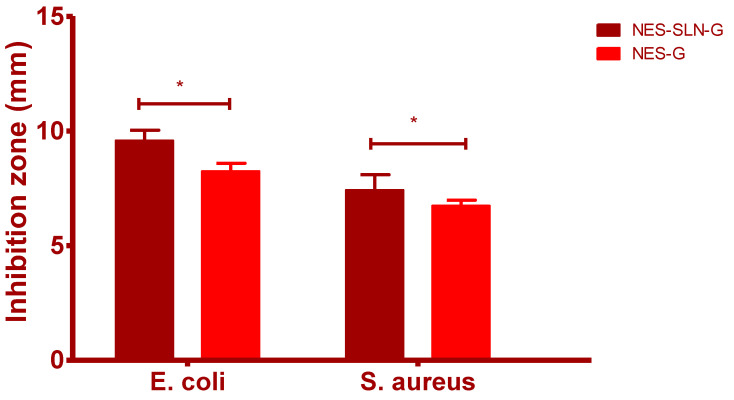
In vitro antimicrobial activity assessment of NES formulations (Avg ± S.D; *n* = 3, * *p* < 0.05).

Different Kolliphor-407 P gel formulations containing NES-SLN and NES were tested for antibacterial efficacy against *S. aureus* and *E. coli*. After 24 h of treatment, the Kolliphor-407 P gel containing NES-SLN had more significant antibacterial activity against *S. aureus* than the pure NES gel (*p* < 0.05) (Figure 7). Recent investigations [1,15] have demonstrated similar antibacterial activity against *S. aureus* and *E. coli* based on determining the zone of inhibition. The findings mentioned above revealed that after 24 h of treatment, the NES-SLN-encapsulated gel had improved antibacterial activity against *S. aureus*.

### 2.6. Confocal Laser Scanning Microscope Study

**Figure 8:**
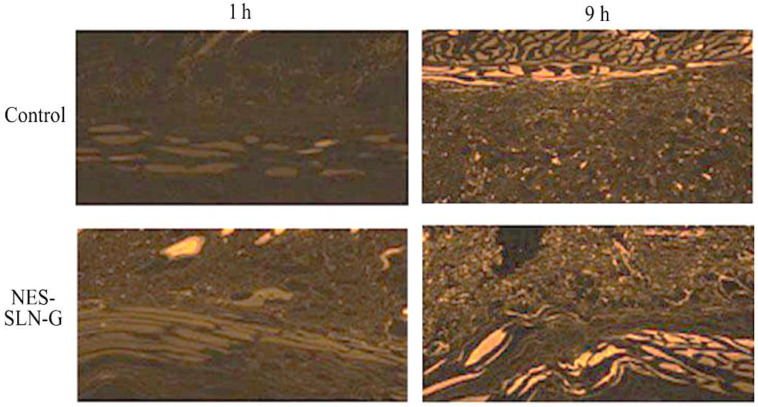
CLSM images for rhodamine-loaded control and NES-SLN-G formulation.

This investigation aimed to see how far the prepared NES-SLN-G could penetrate the skin. Figure 8 shows the CLSM after applying transdermal films containing either pure rhodamine (control) or fluorescence-labeled NES-SLN-G. Compared to the control, the NES-SLN-G carrier was widely dispersed with vigorous fluorescence intensity throughout the mucosal membrane layers. The degree of fluorescence at the end of 9 h reflects the excellent distribution of NES into deeper skin layers.

These findings imply that compounded SLN-G has the potential to improve medication bioavailability by improving NES mucosal membrane penetration deep into the dermal region. Furthermore, because no discrete pores were detected, the permeation was thought to be transcellular. Because the produced particles are lipid-based, their penetration favors direct passage through the phospholipid-based cell membrane.

### 2.7. Stability Studies

The stability of the gel formulation, including MTZ SLNs, was assessed by measuring particle size and in vitro drug release from HEC gels maintained at room temperature (25 °C, 60% RH) for one month. After 1 month of storage, the particle size of NES-SLN in the gel formulation remained stable [197.3 nm] compared to the initial content [196.5 nm]. As evidenced by the f2 value of 82.54, the release profiles of the gel formulations followed the same trends over time (which is more than 50). The inclusion of NES-SLN in the gel formulation helped its stability over a one-month storage period, according to the stability study results.

## 3. Materials and Methods

### 3.1. Materials

NES was purchased from India-based Yarrow Chemicals, Mumbai. Sigma-Aldrich Co. provided stearic acid, Pluronic F 68, and Glycerol monostearate (St. Louis, MO, USA). All additional compounds and solvents used were of analytical quality and were not purified further.

### 3.2. Compatibility Studies by FTIR

To look for important interactions, pure drug and excipients were analyzed using an FTIR spectrometer (Shimadzu IR Affinity-1, Digital IR Spectrometer, Tokyo, Japan) using the KBr pellet method in a range between 4000–450 cm-1 to identify the interactivity [29,30].

### 3.3. Formulation of SLNs of NES (NES-SLN)

The modified solvent injection approach [31] was used to make solid lipid nanoparticles. In 15 mL of dichloromethane, lipids (Stearic acid and Glycerol monostearate) of various concentrations, as well as soya lecithin (0.04% *w*/*v*) and NES (0.5%), were dissolved. This solution was injected at a rate of 1 mL/min using a 0.45 mm/26 G (internal diameter) needle into 100 mL of aqueous phase (purified water) containing 0.25 g of non-ionic surfactant Pluronic F 68 [P-F68] at room temperature (25 °C) under continuous stirring (2000 rpm for 2 h). After that, the solvent was evaporated for 5 h in a vacuum oven at 40 °C and 200–400 mbar.

To investigate the interaction of independent variables, the Box–Behnken design was chosen. The percentages of Stearic acid A (X1), Glycerol monostearate (X2), and P-F 68 were chosen as the three factors (X3). These variables’ effects on particle size (Y1) and entrapment efficacy (Y2) were investigated. Table 6 shows the summary of variables for all 17 runs that were forecasted. Using Design Expert-12 edition, the correlation between the selected factors and responses was further investigated using a statistical analysis strategy and regression equation (Stat Ease, Inc., MN, USA). The SLN formula was tweaked to obtain the smallest particle size and greatest EE. To analyze and optimize the interaction effects of each level on formulation characteristics, a total of 27 experimental runs were necessary. Each trial’s reaction (Yi) was measured using multiple factorial regression analysis (quadratic model).

### 3.4. Characterization of NES-SLN 

#### 3.4.1. Determination of Particle Size

Photon correlation spectroscopy (PCS) was used to measure the particle size (Z-average mean) of prepared SLNs, and each measurement was performed in triplicate using a Zetasizer (Malvern Master Sizer 2000, Worcestershire, UK).

#### 3.4.2. Entrapment Efficacy (EE)

EE is the ratio of the absolute theoretical amount of drug entrapped in the polymer to the practical amount entrapped in the polymer [32,33,34]. Formulations were centrifuged at 25,000 Xg for half an hour, and the supernatant was tested for free NES by measuring the absorbance at 304.8 nm (REMI centrifuge, C-24 BL, Maharastra, India). The total and free amounts of NES were substituted in the following calculation to determine EE.

(3) $$\;\mathrm{EE}\left(\%\right)=\frac{{\mathrm{RP}}_{T\;}-{\mathrm{RP}}_{F}}{{\mathrm{RP}}_{T\;}}\times 100$$

where RP_T_ = total amount of NES used in the preparation of nanoparticles, and RP_F_ = free NES present in the supernatant

### 3.5. Validation and Standardization of Optimization Results

The responses generated by all of the preparations were triggered using Design-Expert software. The responses were used to create the methodology for the study and the response surface graph. A numerical standardization method was used to build an upgraded preparation with a stated minimal and maximal limit for each parameter. The findings were combined to create a desirability function [35,36]. The collection of alternatives was ranked from most desirable to least desirable, and the solutions that met the requirements were noted. The response surface graph revealed the relationship between the independent and dependent parameters. ANOVA was used to investigate the impact of various factors on the slope coefficients. The relative uncertainty was calculated as part of the design validation process by comparing expected and experimental values.

### 3.6. Preparation of Gel (NES-SLN-G)

Kolliphor-407 P (15% *w*/*w*) was used as a gelling agent to formulate NES-SLN-based gel after optimizing the selected variables [37]. Kolliphor-407 was directly added to the NES-SLN dispersion and was allowed to soak overnight, resulting in gel formation. Similarly, the conventional gel was prepared by adding both APIs to an aqueous 15% *w*/*w* Kolliphor-407 P dispersion for comparative study (NES-G).

### 3.7. Physicochemical Characterization of Gel

The SLN colloidal dispersions were tested for size and size distribution (PDI), surface charge, morphology, density, surface tension, viscosity, pH, and spreadability to anticipate their appropriateness for topical application.

#### 3.7.1. Particle Size and Polydispersity Index

At 25 °C, the particle size and particle size distribution were investigated using a Zetasizer (Malvern Instruments, Worcestershire, UK). In brief, 10 L of the material was mixed well with 1 L of deionized water and vortex-mixed for 2 min, and photon correlation spectroscopic analysis was performed as previously described [38]. The average particle size and PDI values were tallied after the findings were recorded in triplicate.

#### 3.7.2. Zeta Potential

A Zetasizer was used to assess the surface charge of manufactured SLN colloidal dispersions (Malvern Instruments, Worcestershire, UK). As previously stated, an aliquot of 700 L of non-diluted colloidal dispersion was supplied to the Zeta potential cell, and its charge was analyzed in triplicate.

#### 3.7.3. Structural Analysis

SEM was conducted to study the morphology features of the optimized microsphere formulation (Jeol JSM-6350, Tokyo, Japan) [38]. The samples were placed onto an aluminum stump after coating for 70 s. Then, stumps were varnished with gold–palladium under argon air with a module in a high-vacuum evaporator. Further, the glazed samples were arbitrarily scanned, and images were recorded [39].

#### 3.7.4. Density

At 25 ± 1 °C, the density of produced SLNs was measured using an ordinary pycnometer. The weight of the empty pycnometer of known volume (V) was first recorded as W1, and the weight of the water-filled pycnometer was recorded as W2 (W2). W3 was the weight of the pycnometer loaded with SLNs. The following equation (5) was used to calculate the density of SLNs [40].

Density of water = W2/V(4)

Density of SLNs = W3/V(5)

Specific gravity of SLNs = (SLNs density)/(Water density)(6)

#### 3.7.5. Surface Tension

The surface tension of the SLN dispersions was measured using a laboratory stalagmometer. The instrument was cleaned with deionized water before being filled with double-distilled water up to point A. The full water was then run down to point B, drop by drop, while the number of drops was counted. For the SLN dispersions, the same methods were followed. As noted in the previous section, the density of SLNs had already been computed. Following that, the surface tension of SLNs was computed using Equation (7) [37].

^γ^1 = (^δ^1 × n1 ÷ ^δ^2 × n2) × ^γ^2 (7)

where:

^γ^1: Surface tension of dispersion;
^γ^2: Surface tension of water;
^δ^1: Density of dispersion;
^δ^2: Density of water;
n1: Number of drops of SLNs;
n2: Number of drops of deionized water.

#### 3.7.6. Viscosity

A viscometer (Model RVT, Brookfield Engineering, Middleboro, MA, USA) with a Helipath stand was used to measure the viscosity of the NES-SLN-G and NES-G formulations. The sample was placed in a beaker and allowed to equilibrate for 5 min before using a T-C spindle at 0.5, 1, 2.5, and 5 rpm to measure the dial reading. At room temperature, the measurements were repeated in duplicate [41].

#### 3.7.7. Determination of pH and Spreadability

A digital pH meter was used to determine the pH of the prepared gels (EQ 610, Equip tronic Mumbai, India). Spreadability (S) was determined by placing 2.5 g over one of the slides, which was then covered with a second glass plate. For 5 min, a weight of 1000 g was permitted to rest on the upper glass plate. After removing the weight, the distance traveled by the upper slide was computed, and spreadability was estimated using the formula.

S = ML/T

where:

S = Spreadability of gel,
M = Weight (g) applied on the upper plate,
L = Length (cm) of the glass plates,
T = Time taken for plates to slide the entire length

### 3.8. Ex Vivo Permeation Study

Drug release from the developed SLNs and their related gels was determined using a Franz diffusion cell. A 0.45 m cellulose acetate membrane was attached between the donor and receptor compartments [42,43]. To imitate skin conditions, the receptor compartment was filled with acetate buffer (pH 5.5) at 32 ± 2 °C. Magnetic stirring was set to 600 rpm to retain the sink condition. One gram of the material was supplied to the donor compartment. At 0, 0.5, 1, 2, 4, 8, 16, and 24 h, an aliquot of 0.5 mL was taken from the receptor compartment through the sampling port using a calibrated syringe. A UV–visible spectrophotometer (UV-1601 Shimadzu, Kyoto, Japan) was used to examine the samples at a maximum wavelength of 304.8 nm. The standard calibration curve was used to calculate the cumulative release of tacrolimus from the proposed carrier system. All of the data were calculated as the average of three experiments [44].

### 3.9. In Vitro Antimicrobial Activity Assessment

The antibacterial activity of the produced gels was tested using Mueller–Hinton agar plates and the plate diffusion method. Gram-positive and Gram-negative bacteria were employed, namely, *Staphylococcus aureus* ATCC 29,213 and *Escherichia coli* ATCC 25922. Fresh cultures of both bacteria were injected onto the plates at an optical density of 108 bacteria/mL (equal to 0.5 McFarland turbidity) in a sterile 0.9 percent salt solution. Each well (6 mm in diameter) was made by removing the agar with the back of a sterile Pasteur pipette after drying at room temperature for 15 min. Each well received the produced gel formulations containing NES or NES SLNs (0.1 g). The plates were then incubated for 24 h at 37 degrees Celsius. Each assay was carried out three times. The inhibition zones were measured with a 0.1 mm precision caliper (RS PRO, Shanghai, China). As a control, the Kolliphor-407 P gel formulation without NES was employed [45].

### 3.10. Confocal Laser Scanning Microscope (CLSM) Study

The drug was substituted with rhodamine at a 0.15 mol/mL concentration in NES-SLN-G, as described before. Rhodamine is a group of related dyes that are a subset of the triarylmethane dyes and are xanthene derivatives [46]. Dialysis extracted unentrapped rhodamine using a dialysis bag (Sigma Aldrich, St. Louis, MO, USA; 14 kDa). A CLSM (Olympus FV10i, M/s Olympus Singapore Pte Ltd., River Valley Road, Singapore) was used to examine the presence of fluorescence in the various mucosal membrane layers after 1 and 9 h of treatment with the fluorescence-labeled SLN formulation.

### 3.11. Stability Study

The physical and chemical stability of the Kolliphor-407 P gel containing NES SLNs was assessed by quantifying the particle size of NES SLNs in the Kolliphor-407 P gel, as well as the in vitro drug release over a month of storage at room temperature (25 °C, 60% RH) [47].

## 4. Conclusions

This research looked at the adaptability of QbD in the lab and provided high-level suggestions on the scope and applicability of QbD. The research was carried out to formulate solid lipid nanoparticles, and the integration of the QbD concept with modern methodologies was confirmed to be a significant advancement for the pharmaceutical industry since this time- and cost-saving process ensures a high-quality result. The Box–Behnken design was used to successfully optimize NES-SLNs. According to the desirability approach, a formulation having 0.467 percent stearic acid, 0.275 percent glyceryl monostearate, and 1.23 percent P-F 68 can achieve the optimal formulation parameters for NES-SLN preparation. The relative inaccuracy was found to be within acceptable bounds, validating the design’s correctness. Particle size, PDI, and zeta potential were assessed after the improved formulation was successfully manufactured in gel systems. SEM analysis indicated that the lipid nanoparticles did not aggregate and that the formulation was dispersed uniformly. In comparison to NES-G and NES-SLN, drug release experiments showed that the NES-SLN-G formulation had a more regulated release. Owing to the enhanced antibacterial activity, the designed gel formulation was proven to be efficacious in treating wounds.

## Figures and Tables

**Table 1:** Projected trial batches and their responses for central composite design.

	Factor 1	Factor 2	Factor 3	Response 1	Response 2
Run	A:Stearic Acid	B:Glycerol Monosteratae	C:P-F 68	Particle Size	EE
	(%)	(%)	(%)	nm	%
12	0.3	0.3	1	354	78
6	0.3	0.2	1.5	398	79
5	0.3	0.1	1	223	84
13	0.3	0.2	0.5	219	85
2	0.4	0.1	0.5	299	72
16	0.4	0.3	0.5	340	76
4	0.4	0.1	1.5	346	79
1	0.4	0.3	1.5	342	79
3	0.4	0.2	1	265	82
7	0.4	0.2	1	264	83
9	0.4	0.2	1	263	83
14	0.4	0.2	1	263	84
17	0.4	0.2	1	266	84
10	0.5	0.1	1	288	76
11	0.5	0.2	0.5	345	77
15	0.5	0.3	1	201	87
8	0.5	0.2	1.5	245	89

**Table 2:** Model summary statistics of selected responses.

	Source	Sequential *p*-Value	Lack of Fit *p*-Value	Adjusted R^2^	Predicted R^2^	
Particle Size	Linear	0.7345	<0.0001	−0.1195	−0.8423	
	2FI	0.0057	<0.0001	0.5634	−0.1454	
	Quadratic	<0.0001	0.0002	0.9730	0.9130	Suggested
	Cubic	0.0002		0.9995		Aliased
EE	Linear	0.5813	0.0012	−0.0644	−0.7290	
	2FI	0.0325	0.0032	0.4025	−0.5804	
	Quadratic	<0.0001	0.4553	0.9639	0.9628	Suggested
	Cubic	0.4553		0.9650		Aliased

**Table 3:** Model (quadratic) fit summary of the responses.

Parameter	PS	EE
Std. Dev.	9.19	0.8494
Mean	289.47	81.00
C.V. %	3.17	1.05
Adeq Precision	27.5567	26.0962
**Lack of Fit F-value**	114.46	1.07
**Lack of Fit *p*-value**	0.0785	0.4553
Model F-value	65.10	48.51
Model *p*-value	<0.0001	<0.0001

**Table 4:** ANOVA coefficients for both responses.

	Intercept	A	B	C	AB	AC	BC	A^2^	B^2^	C^2^
**Particle size**	264.2	−14.375	10.125	16	−54.5	−69.75	−11.25	−13.85	16.15	51.4
***p*-values**		0.0031	0.0169	0.0017	<0.0001	<0.0001	0.0441	0.0175	0.0086	<0.0001
**EE**	83.2	0.375	1.125	2	4.25	4.5	−1	2.025	−3.975	−2.725
***p*-values**		0.2519	0.0072	0.0003	<0.0001	<0.0001	0.0507	0.0018	< 0.0001	0.0003

**Table 5:** Comparison of characterization of NES-SLN-G and NES-G. (*—Values are represented as Avg ± S.D (*n* = 3).)

	NES-SLN-G *	NES-G *
**Particle size**	196.5 ± 1.5 nm	542.5 ± 4.2 nm
**PDI**	0.15 ± 0.02	0.58 ± 0.04
**Zeta potential**	−32.5 ± 1.2 mV	6.8 ± 0.45 mV

PDI—Polydispersity index.

**Table 6:** Box–Behnken design (experimental plan of mixture design (component levels and selected response)) for optimization of SLN of NES.

Component	Level		Response	Constraints
	Low	High		
Stearic acid (%); (X_1_)	0.3	0.5	Particle size (Y_1_)	Minimum
Glycerol monostearate (%); (X_2_)	0.1	0.3	EE (Y_2_)	Maximum
P-F 68 (%) (X_3_)	0.5	1.5		

## Data Availability

Not applicable.
